# Fahr disease: Idiopathic basal ganglia calcification

**Published:** 2017-01-05

**Authors:** Gholam Ali Shahidi, Mahdi Safdarian

**Affiliations:** ^1^ Department of Neurology, School of Medicine, Iran University of Medical Sciences, Tehran, Iran

**Keywords:** Fahr Disease, Idiopathic Basal Ganglia Calcification, Tomography, X-Ray Computed

A 23-year-old man presented with progressive bulbar and oromandibular dystonia, in addition to distal limbs involvement. In neurological exam, the patient had diverse manifestations, most commonly movement disorder, cognitive impairment, ataxia and speech disorder. Other minor neurologic manifestations included pyramidal signs, psychiatric features, and gait disorders. He had no positive family history, and his parents were not relative. Calcifications in the brain computed tomography scan (CT scan) are seen primarily in the basal ganglia and in other areas such as the cerebral cortex (Figure 1).

Fahr disease (FD) is a rare genetically dominant, neurodegenerative disorder characterized by idiopathic bilateral deposits of calcium in the striopallidodentate area.^1^ Symptoms may include deterioration of motor function, dementia, seizures, headache, dysarthria, spasticity, eye impairments, and athetosis.^2^ After ruling out the medical calcium metabolism abnormalities, FD is diagnosed by the presence of extensive bilateral symmetric intracranial calcifications and developmental defects.

**Figure 1:**
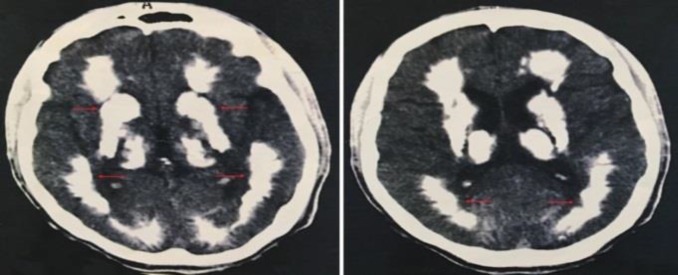
Brain computed tomography scan (CT scan) of the patient with Fahr disease (FD) showing bilateral symmetrical calcifications in the basal ganglia and the cerebral cortex (Arrows)

The major differential diagnosis includes hypoparathyroidism. Bilateral symmetric calcification involving striatum, pallidum, dentate nucleus, thalamus, and white matter is reported from asymptomatic individuals to a variety of neurological conditions. The key point is that there is no known calcium metabolism abnormality in autosomal dominant or sporadic bilateral striopallidodentate calcinosis. Movement disorders, especially Parkinsonism is the most common presentation followed by cognitive impairment and ataxia.^3^

Magnetic resonance imaging (MRI) is sensitive in detecting brain abnormalities; however, it is difficult to identify calcifications by routine MRI because calcifications. Therefore, brain CT scan is considered to be critical for detecting and localizing the extent of intracranial calcifications.^4^

## Notes:

**How to cite this article:** Shahidi GA, Safdarian M. Fahr disease: Idiopathic basal ganglia calcification. Iran J Neurol 2017; 16(1): 53-4.
